# Exploring the optical limiting, photocatalytic and antibacterial properties of the BiFeO_3_–NaNbO_3_ nanocomposite system

**DOI:** 10.1039/d0ra09776d

**Published:** 2021-02-24

**Authors:** Rehana P. Ummer, Sreekanth Perumbilavil, Jiya Jose, Sabu Thomas, Pramod Gopinath, Nandakumar Kalarikkal

**Affiliations:** International School of Photonics, Cochin University of Science and Technology Cochin 682022 India rehana2009spap@gmail.com pramod@cusat.ac.in; Inter University Centre for Nanomaterials and Devices, Cochin University of Science and Technology Cochin 682022 India; Department of Biotechnology, Cochin University of Science and Technology Cochin 682022 India; Department of Applied Physics, Aalto University School of Science P.O. Box 15100 FI-00076 Aalto Finland; School of Pure and Applied Physics, Mahatma Gandhi University Kottayam Kerala 686560 India nkkalarikkal@mgu.ac.in; International and Inter University Centre for Nanoscience and Nanotechnology, Mahatma Gandhi University Kottayam Kerala 686560 India

## Abstract

Thin films of BiFeO_3_–NaNbO_3_ composites were fabricated in a PMMA matrix. XRD and HRTEM were used for structural investigations. The grain size and surface morphology of samples were analysed through HRTEM images. The self-cleaning property of any material accelerates its industrial applications. Hence, along with the optical limiting performance, the photocatalytic and antibacterial activity of BiFeO_3_–NaNbO_3_ composite samples were also studied. BiFeO_3_–NaNbO_3_ films fabricated in the PMMA matrix exhibit strong optical nonlinearity when excited by 5 ns laser pulses at 532 nm. The origin and magnitude of the observed optical nonlinearity were explained on the basis of the weak absorption saturation and strong excited state absorption. The photocatalytic performance of samples was analysed by dye degradation method using Methyl Orange dye. The dye degradation rate in the presence of the catalyst is heeded in a particular time interval, which exhibits the photocatalytic performance of the samples. The destruction of microbial organisms that are in contact with the material was contemplated, which could prove its antibacterial activity. The effect of the particle size on the photocatalytic activity was also investigated.

## Abstract 2

Thin films of BiFeO_3_–NaNbO_3_ composites were fabricated in a PMMA matrix.

## Graphical abstract

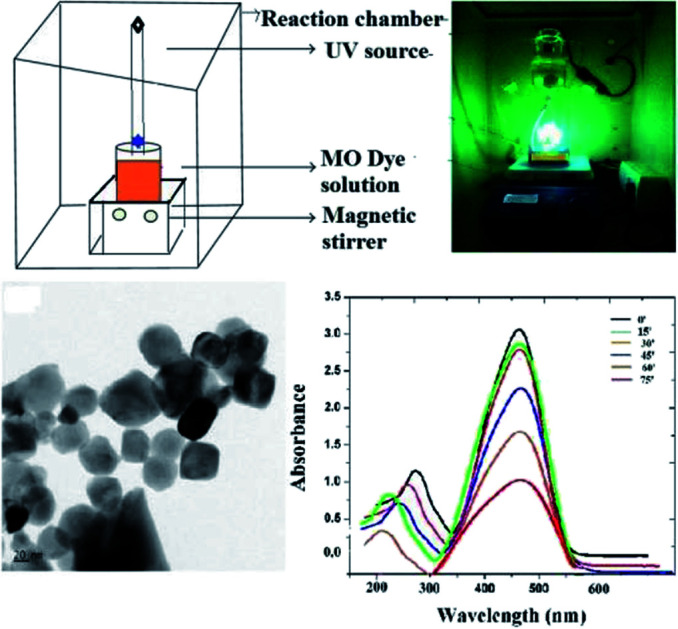

## Introduction

The BiFeO_3_–NaNbO_3_ composite material is a well known multiferroic, which has found wide applications in storage devices and sensors, among others.^1–10^ The energy storing and optical limiting property of this material has been examined previously.^12,13^ In our previous article, we have scrutinized the electric, magnetic, and optical limiting properties of the BiFeO_3_–NaNbO_3_ composite in its powdered form^12^ and thin film in a PVDF-TrFE matrix.^13^

Optical nonlinearities in ferrites are relatively unexplored, and reports^17,18^ are rare compared to organics, semiconductors and metals.^19–22,42–46^ Different phenomena are used for optical limiting, such as light scattering in carbon black suspensions,^1,26^ beam fanning in photorefractive materials,^7^ and nonlinear absorption in absorbing materials (reverse saturable absorption RSA) and in transparent materials (two-photon absorption TPA). Phthalocyanines,^3^ porphyrins,^4^ and carbon C_60_ are efficient RSA materials.^5^ Their efficiency as optical-power limiters occurs for long pulse duration, but they have the drawback of being absorbing and highly colored.^27^ On the other hand, TPA materials are transparent in the visible region at low energy with an instantaneous response and no saturation. For nanosecond pulses, recent results have highlighted the interest of the organic two-photon absorbers.^28–36^ Recent studies give evidence of a very promising nonlinear optical efficiency for BFO, which is found to be superior to the standard oxide materials previously introduced.^23–25^ Optical power limiters are required to protect eyes or photodetectors from damage caused by tunable laser pulses^14–16^

The antimicrobial killing property of any material is a desired one, and it will be an added advantage if we can develop self-cleaning devices. Self-cleaning materials can solve a number of environmental problems, as they disinfect harmful microorganisms from surfaces. Researchers found that materials with photocatalytic and antibacterial activity can solve hospital acquired problems to a certain extent.^8^ This will create a hygienic environment in hospitals and laboratories. Polymer host multiferroic nanocomposites are appropriate for device fabrication, as they are highly flexible and can be casted in thin film form.^9^ Several other problems, such as high leakage current and dielectric loss, can be eliminated in these polymer-based composites.^10,11^ Bismuth compounds were found to be clinically nontoxic,^37^ and reports show that they are active against bacteria, such as *E. coli* and *H. pylori*.^28^ The photocatalytic and antibacterial activity of semiconductor nanomaterials were found to be excellent.^12,13,27^ Various organic pollutants can be degraded completely through photocatalysis using metal oxide semiconductor nanostructures under UV light irradiation.^30,36,39^ They have been used widely in many investigations because of their application for the destruction of chemical contaminants and water splitting. Conventional semiconductor photocatalysts, such as TiO_2_ and ZnO, are cost-effective, non-toxic, and serve as antimicrobial agents.^50–52^ As they have a wide band gap (3.2 eV), they are UV absorbers and consist only 4% solar light irradiation, which is one of their practical application hindrances.^27,30–32^ Hence, research on developing a metal oxide nanoparticle with a narrow band gap is of current interest.^30–32^

## Experimental

### Preparation of the BiFeO_3_–NaNbO_3_ powder

Preparation techniques of the composites are as follows.^12^ The solid solution ceramics of a (1 – *x*)BiFeO_3_–*x*NaNbO_3_ composite system for different *x* values (*x* = 0, 0.1, 0.5, and 0.7) were prepared by modified sol–gel method called pechini method.^24,25^ AR grade ammonium niobate oxalate (Sigma-Aldrich >99% pure), Bi(NO_3_)_3_ (Sigma-Aldrich >99% pure), Fe(NO_3_)_3_ (Sigma-Aldrich >99% pure), and NaNbO_3_ (Sigma-Aldrich >99% pure) were used as raw materials. First, the raw materials were carefully weighed in a stoichiometric ratio, dissolved in a citric acid aqueous solution (in a 1 : 1 molar ratio with respect to the total metal cation), and the pH value was adjusted to 5 using NH_3_OH. The clear solution thus obtained was dried at 100 °C to form the gel, and the gel was burnt at 500 °C to get the ceramic powders. After that, the powder was ground and pressed into pellets. Finally, the pellets were sintered at 850 °C for 1 hour to get the final sample.

### Preparation of BiFeO_3_–NaNbO_3_–PMMA composite films

Solvent casting was opted for making the film sample, as it is simple and less expensive compared to other methods. The BiFeO_3_–NaNbO_3_-PMMA ceramic powder prepared by Pechini method (5 wt%) was dispersed in PMMA solution, and then ultrasonicated for 30 min. A gelatinous brownish white solution was obtained. The obtained solution was poured in to Petri dishes, and kept at room temperature for 2–3 days. Within this time, the desired films were formed.

The crystal structures of the samples were examined by Phillips X'Pert Pro XRD with Cu-Kα radiation (1.54056 Å). Step-scanned powder XRD data were collected in the 2*θ* range of 10°–80° at room temperature on the finely ground sample. The detailed structural analysis was performed using a scanning electron microscope (JEOL JSM 6390) and transmission electron microscope (JEOL JEM 2100). In order to determine the reduction activity of the sample, the photocatalytic reduction of Methyl Orange (MO) was investigated. The antimicrobial activity of the powder samples was assessed using the well diffusion method.

## Results and discussion

### Structural analysis

**Fig. 1:**
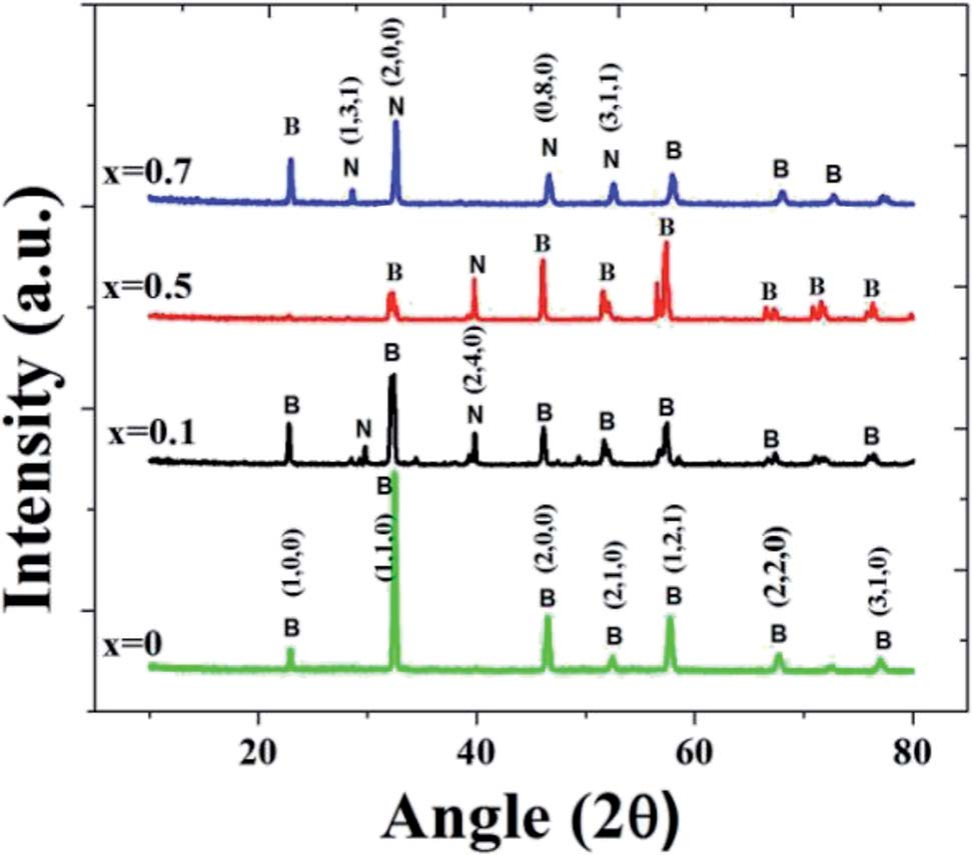
XRD patterns of (1 − *x*)BiFeO_3_–*x*NaNbO_3_ composites.

Structural analysis was carried out on BiFeO_3_–*x*NaNbO_3_ powder samples. For the device fabrication, it is desirable to incorporate the material in some polymer matrix. Hence, we have prepared BiFeO_3_–NaNbO_3_ PMMA composite films. PMMA was chosen as it is less expensive and a biocompatible polymer. The X-ray diffraction pattern of the (1 − *x*)BiFeO_3_–*x*NaNbO_3_ composite system for different *x* values at room temperature are shown in Fig. 1. In the XRD spectra, B and N represent the BiFeO_3_ and NaNbO_3_ phases, respectively. The absence of non-perovskite phases, such as Bi_2_Fe_4_O_9_ and Bi_2_O_3_/Fe_2_O_3_, is an achievement of the pechini method, as it is a common occurrence when we adopt other synthesis techniques. The crystal structure was found to be changing from rhombohedral to orthorhombic by increasing the amount of the NaNbO_3_ phase in the composite.^38–40^

It was observable from the XRD spectra that the peaks corresponding to NaNbO_3_ appear when *x* increases. Similarly, the BiFeO_3_ phase decreases gradually with increasing *x* value. The crystallite size was calculated using the Scherrer's equation from the full width at half maximum (FWHM) of the diffraction peaks. The average grain size values calculated from the Scherrer equation are 6.2 nm, 10.9 nm, 41.7 nm and 79.2 nm for *x* = 0, *x* = 0.1, *x* = 0.5 and *x* = 0.7, respectively. As the *x* value increases, the plane corresponding to (110) (which represents BFO) shifts to the right side, which represents the (200) plane of NaNbO_3_ (JCPDS cards JCPDS 74-2016 and JCPDS 89-8957).

**Fig. 2:**
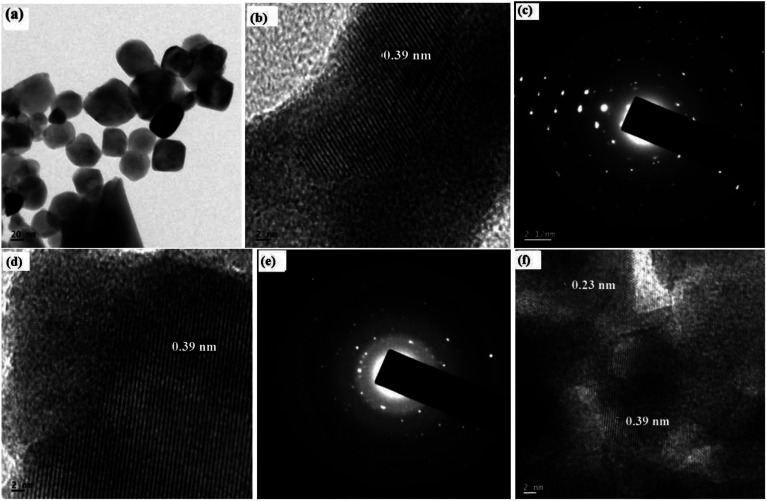
(a) TEM image of *x* = 0, (b) HRTEM image of *x* = 0, (c) SAED pattern of *x* = 0, (d) HRTEM image of *x* = 0.1, (e) SAED pattern of *x* = 0.1, (f) HRTEM image of *x* = 0.5.

Transmission electron microscopic (TEM) images of the samples are shown in Fig. 2. The average particle size was found to be 50 nm. TEM pictures show that there is a slight agglomeration due to moisture absorption. The SAED pattern confirms the polycrystalline nature of the samples. The SAED patterns show the presence of sharp diffraction spots, which is a clear indication of well developed, crystalline nanoparticles. The lattice spacing (*d*) was calculated from the HRTEM images, and it was found to be matching with the JCPDS values corresponding to BiFeO_3_ and NaNbO_3_. The *d* spacing of 0.23 nm and 0.39 nm corresponds to the (221) plane of orthorhombic NaNbO_3_ (JCPDS 89-8957) and the (100) plane of rhombohedral BiFeO_3_ (JCPDS 74-2016), respectively. By increasing the *x* component, we could not see much difference in the TEM images. However, in Fig. 2(f), we can see that two different planes are observable. This kind of HRTEM image represents the composite materials, which contain more than one phase.

The band gap values for each composite was studied, which is detailed in our previous article.^12^ The band gap values are 1.7 eV, 1.9 eV, 2.3 eV, and 2.8 eV for *x* = 0, *x* = 0.1, *x* = 0.5 and *x* = 0.7, respectively,^12^ which is consistent with other reported values.^39^ The absorption cut-off wavelength of the as-prepared composite samples lies between 500–600 nm, which is close to the reported value for pure BFO (*i.e.*, 560 nm),^40^ suggesting that the present material can absorb visible light in the wavelength range of 400–565 nm.

**Fig. 3:**
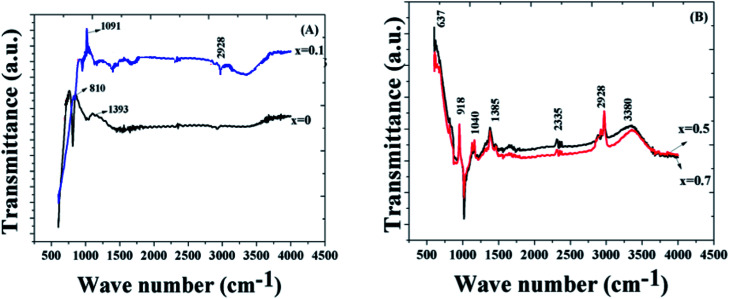
(a) FTIR analysis of the samples for *x* = 0 and 0.1. (b) FTIR analysis of the samples for *x* = 0.5 and 0.7.

Fig. 3 shows the FTIR spectra of different compositions of the samples. The band at 637 cm^−1^ corresponds to the bending modes of the vibration of bismuth oxide,^53^ and the band at 810 cm^−1^ is due to the Fe–O bond, which indicate the highly crystalline BiFeO_3_ phase. The peak at 1091 cm^−1^ may be due to the C–C bond. It is probable that some of the hydrated carbonates detected in the high temperature FTIR samples are a consequence of a reaction that occurs between the powders after thermal decomposition with moisture and carbon dioxide in the air during sample storage prior to recording the FTIR spectra.^54^ The broad absorption bands from 3000 cm^−1^ to 3600 cm^−1^ arose from the antisymmetric and symmetric stretching of the bonds from the H_2_O and OH-1 groups. As we have sintered the sample at high temperature (>800 °C), this band corresponds to the absorption of water from the environment.^55^ The bands at 1040 cm^−1^ and 1385 cm^−1^ indicate the existence of nitrate ions.^56^ A peak at 2335 cm^−1^ is representative of the nitrile group, and the band at 2928 cm^−1^ is due to the C–H stretching vibrations.^57^

### Optical limiting studies

The optical limiting studies were carried out on BiFeO_3_–NaNbO_3_ ceramics embedded PMMA film composites. The open aperture *Z*-scan technique was employed to investigate the nonlinear optical properties of the as-prepared films. The open aperture *Z*-scan study of the BiFeO_3_–NaNbO_3_ powder samples was reported in our previous article.^12^ The intensity-dependent transmission was measured using an automated open aperture *Z*-scan setup. A laser beam was focused using a planoconvex lens (focal length = 10.75 cm). The samples were mounted on a programmable linear translation stage. The linear transmittance of the samples are 69% and 52% for *x* = 0 and *x* = 0.1 at the excitation wavelength (532 nm), which is then translated symmetrically along the beam axis (*z*-axis) through the focus. At each position (*z*), the sample experiences a different laser fluence/intensity, with the maximum fluence/intensity at the focus. The transmission corresponding to each position (toward and away from the focus) was measured using a pyroelectric laser probe (Rjp 735, Laser Probe, Inc.), and the data were recorded. Thus, the dependence of transmission on the fluence/intensity at different positions will essentially show the nonlinear absorption of the material. A detailed experimental description can be found elsewhere.^41^ The data obtained were plotted against the sample position, from which the nonlinear optical parameters can be calculated by numerically fitting them to the transmission equations.

**Fig. 4:**
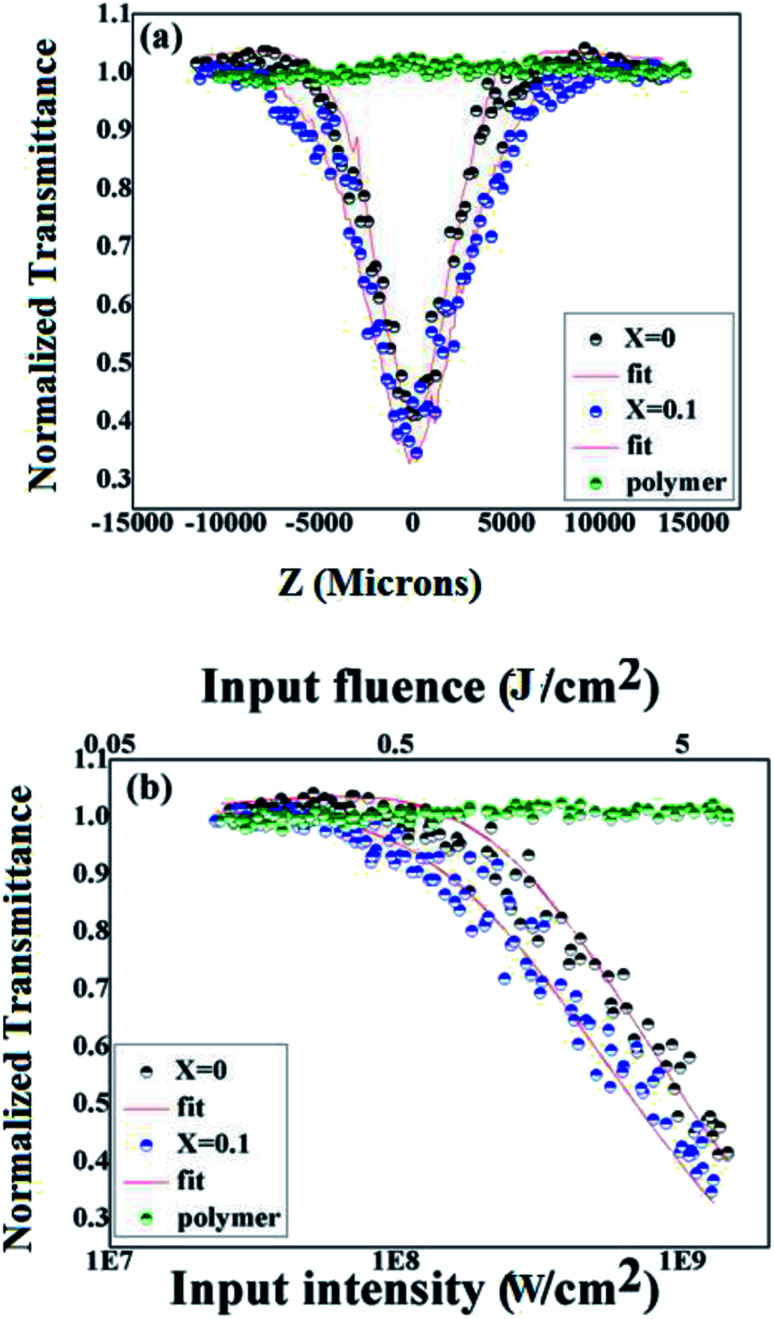
(a) Open aperture *Z*-scan curves obtained in PMMA, *x* = 0 and *x* = 0.1 compositions for 5 ns pulse excitation. (b) Intensity-dependent normalized transmission in PMMA, *x* = 0 and *x* = 0.1 compositions for 5 ns pulse excitation.

The *Z*-scan and fluence/intensity-dependent transmission curves of the films using 5 ns laser pulses at 532 nm are shown in Fig. 4. The input laser pulse energy was fixed to 25 μJ. The *Z*-scan traces show decreased transmittance at the higher laser fluences/intensity, indicating the occurrence of nonlinear absorption. The nonlinear optical coefficients were calculated by numerically fitting the obtained *Z*-scan data to standard nonlinear transmission equations. It is well known that the nonlinear optical transmission of a medium can have contributions from saturable absorption (SA) and/or reverse saturable absorption (RSA). The occurrence of RSA is material-dependent, and can happen due to phenomena, such as excited state absorption (ESA), free carrier absorption (FCA), two-photon (2PA) or three-photon (3PA) absorption and nonlinear scattering. As the excited state absorption is the main process leading to nonlinear absorption at the excitation pulse width of 5 ns, ESA will be a maximum resulting in strong RSA and the contribution of 2PA will be relatively small. Considering this fact, we numerically modelled the results obtained from the *Z*-scan for the nonlinear transmission equation involving the excited state absorption cross-section, and the saturation fluence can be written as:^14,43^1
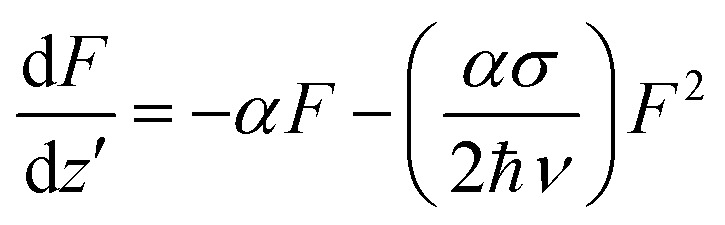
where *σ* is the excited state absorption cross section, *F* is the input laser fluence, *z*′ is the sample path length, ℏ is the Planck constant, and *ν* is the laser frequency given by *c*/*λ*, where *c* is the light velocity and *λ* is the wavelength. The absorption coefficient *α* is given by *α*(*F*) = *α*_0_/1 + *F*/*F*_s_, where *F*_s_ is the saturation fluence. Since *F*_s_ is given by *hν*/2*σ*_0_, it is possible to calculate *σ*_0_, the ground state absorption cross-section, from the value of *F*_s_. The nonlinear transmission behaviour will depend on the values of *σ*/*σ*_0_ and *F*_s_. The value of *σ*/*σ*_0_ gives the figure of merit for a nonlinear optical material. The calculated nonlinear optical parameters are presented in Table 1.

**Table 1:** Nonlinear optical parameters

Sample	Linear transmission at 532 nm (%)	Laser pulse energy (μJ)	Excited state absorption cross section, *σ* (cm^2^)	Saturation fluence, *F*_s_ (J cm^−2^)	Ground state absorption cross section, *σ*_0_ (cm^2^)	*σ*/*σ*_0_
PMMA	92	25	—	—	—	—
*x* = 0	69	25	3.1 × 10^−19^	10	1.9 × 10^−20^	16.5
*x* = 0.1	52	25	3.4 × 10^−19^	12	1.6 × 10^−20^	21.9

As shown in Fig. 4, the PMMA film does not show any sign of nonlinear absorption. Therefore, the optical nonlinearity observed in the composite films was completely from the ceramic nanoparticles. The incorporation of NaNbO_3_ into BiFeO_3_ increases the nonlinear absorption in the composite films. For understanding the optical limiting efficiency of the material, their optical limiting threshold (OLT) value is an important parameter. The OLT of an optical limiter is the value of the input fluence at which the transmittance falls to 50% of the linear transmittance. In the present case, the OLT values were found to be 5.1 J cm^−2^ and 3.38 J cm^−2^ for *x* = 0 and *x* = 0.1, respectively. A lower OLT value indicates higher limiting efficiency. Compared to other recent reports, our material was found to have higher optical limiting property.^45–47^ However, the optical limiting property of the BiFeO_3_–NaNbO_3_ ceramic powder is higher than that of the composite film.^12^

### Photocatalytic study

**Fig. 5:**
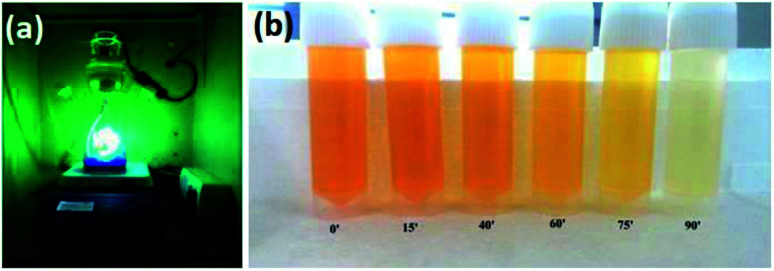
(a) Photograph of the photocatalytic unit. (b) Colour change of the MO dye solution with *x* = 0.1 catalyst of 10.9 nm size in a particular time interval.

The photocatalytic oxidation activities of the samples were evaluated by the degradation of methyl orange (MO). Fig. 5(a) shows the photograph of the photocatalytic setup, while the reaction is going on. The photocatalytic experiments were examined by adding the composite powder to a quartz tube. 0.1 g L^−1^ of the catalyst was added to 100 mL of 10 mg L^−1^ MO solution. Before turning on the UV lamp, the suspension was located in the magnetic stirrer under dark for 1 h to reach an adsorption–desorption equilibrium. During the UV irradiation, 3 mL of the suspension solution was extracted at every 15 minute interval, and then filtered to remove the catalysts from the solution. The MO concentration in the filtrated solution was determined using a UV–Vis spectrophotometer (UV-9600).

The degradation of Methyl Orange dye in the presence of the catalyst material under UV light is shown in Fig. 5(b). MO showed a major absorption band at 484 nm, corresponding to the big conjugate structure of the benzene ring and naphthalene ring surrounded by –N=N–, which is the colour development group in the visible region. In the photo decolouration process, the major absorption band (484 nm) has decreased gradually with elapsing time. The apparent decrease of the absorption band at 484 nm indicated that BiFeO_3_ had initiated the photodegradation, and made the band break and decolorize.

**Fig. 6:**
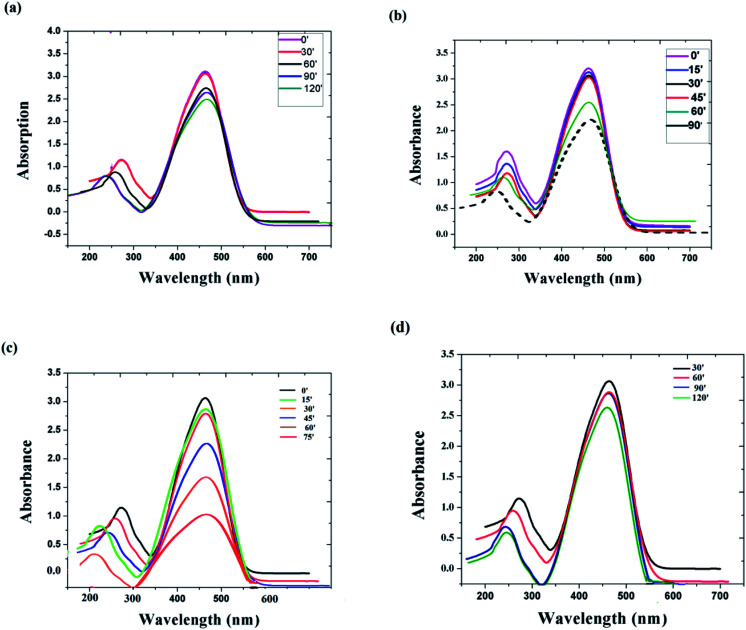
Absorbance changes of MO under the photocatalytic process with (a) *x* = 0 catalyst of 135 nm size, (b) *x* = 0 catalyst of 6 nm size, (c) *x* = 0.1 catalyst, (d) *x* = 0.5 catalyst.

In order to check the influence of the particle size on the degradation efficiency of the catalyst, we initially used the same catalyst material of two different sizes. Fig. 6(a) and (b) represent the photo decolouration of MO catalyzed by *x* = 0 composition of 135 nm size and 6 nm size, respectively. After each 30′, we measured the UV-absorption spectrum. Even though the dye degradation occurs in both cases, the reaction rate is higher for the 6 nm-sized particles. We have measured the degradation efficiency of different compositions (*x* = 0, *x* = 0.1 and *x* = 0.5), and it is manifested. Fig. 6(c) and (d) represent the photo decolouration of MO catalyzed by *x* = 0.1 and *x* = 0.5, respectively. The reaction rate is higher for the *x* = 0.1 composition. By increasing the NaNbO_3_ content (Fig. 5(d)), the catalytic property decreases. The photograph of the colour change of the MO solution after photocatalysis is shown in Fig. 5(b). Within 3 hours of time, there is a considerable change in the colour of the solution.

It is well known that the surface morphology of a photocatalyst can strongly affect the photocatalytic activity since the photocatalytic reaction occurs on the surface. HRTEM and other structural studies have revealed that the present samples mainly contain a number of nano sized and micron sized particles on the surfaces. Obviously, the enhancement of the photocatalytic activities can be attributed to the increase in the surface area of the samples. On the other hand, a decrease in the particle size also leads to a larger surface area, and thus increases the available surface active sites.^23^

At higher NaNbO_3_ concentration (*x* = 0.5), it was found that the dye degradation is a slow process. The experiment was repeated with NaNbO_3_ alone, and it was found that the absorption intensity shows a considerable decrease after 24 hours. We also found out that by increasing the nanoparticle concentration in the dye solution, the photocatalytic property was reduced. It is due to the agglomeration of the nanoparticle, which might act like bulk materials.

**Fig. 7:**
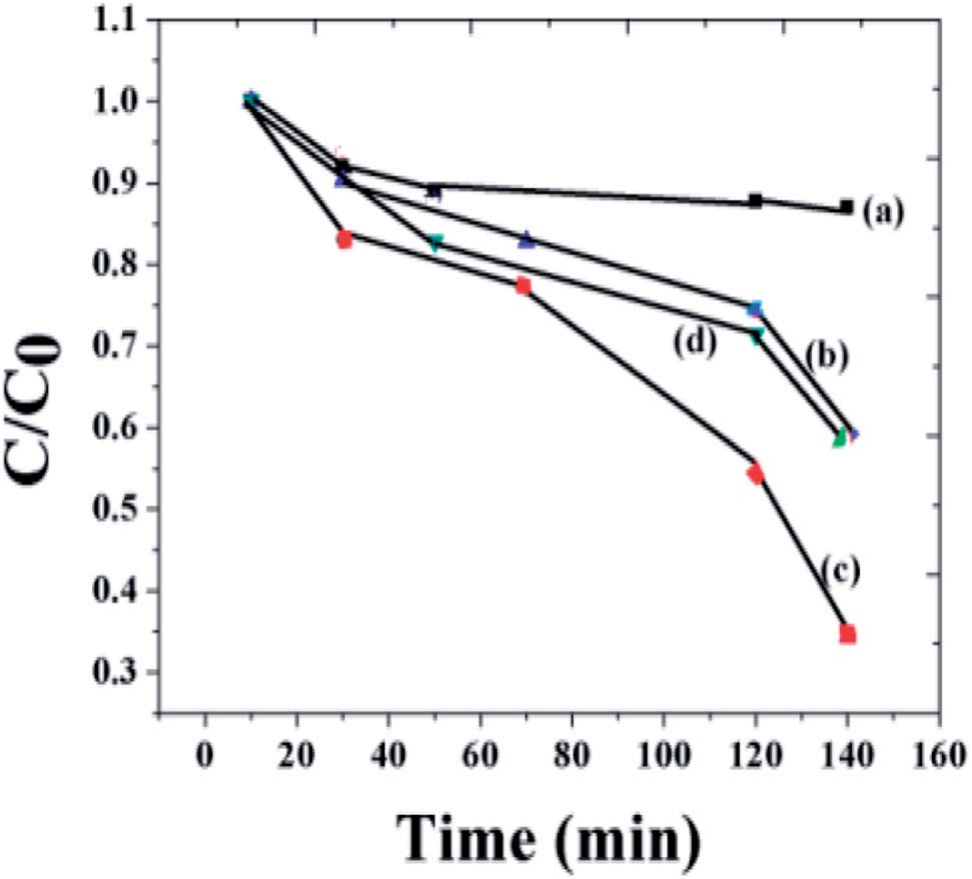
The degradation process of methyl orange by different photocatalysts: (a) *x* = 0 composition with 135 nm size, (b) *x* = 0 composition of 6 nm size, (c) *x* = 0.1 composition, (d) *x* = 0.5 composition.

In order to quantitatively compare the degradation rate, the pseudo-first-order kinetic curves of the MO photodegradation were also plotted. Fig. 7 displays the degradation process of MO by different photocatalysts. It is clear from the figure that the rate of the degradation activities of the *x* = 0.1 composition is higher than that of pure BFO. The rate of the photocatalytic activity of the composite has reduced remarkably with the increase of NaNbO_3_. By decreasing the concentration of the nanoparticle, the rate of the reaction was found to increase.

### Antibacterial study

Gram-positive *Staphylococcus aureus* (*S. aureus*) and Gram-negative *Escherichia coli* (*E. coli*) were used to evaluate the antibacterial characteristics and bactericidal efficacy of the nanoparticles.

### *Escherichia coli* (*E. coli*)

*E. coli* is a Gram-negative bacterium of enteric origin belonging to the family Enterobacteriaceae, and is commonly seen in soil, water, animals, humans and sewage. It has a diameter of 1.1–1.5 μm and length of 2.0–6.0 μm. 10–20% of the Gram-negative cell wall compositions are peptidoglycan, phospholipids, lipoproteins, lipopolysaccharides and proteins composed of the 7 nm thick outer membrane of their cell wall. The normal temperature range for *E. coli* is 21–37 °C and pH 4.4–9.0 with an optimum pH of 6.0–7.0. Out of the numerous different strains of this faecal coliform bacteria, only some are harmful. This bacterium is selected as an indicator organism of faecal contamination since they generally live longer than pathogens. They are found in greater numbers, and are less risky to collect or culture in a laboratory than pathogens. Monitoring for these indicator organisms is an easy and economical method to assess the bacterial contamination of water.

### *Staphylococcus aureus* (*S. aureus*)

*S. aureus* is a Gram-positive coccal bacterium of the family Firmicutes, and is frequently found in the human respiratory tract and on the skin. Hence, it is estimated that 20% of the human population are long-term carriers of *S. aureus*. Although *S. aureus* is not always pathogenic, it causes a range of illnesses from minor skin infections such as pimples, impetigo, boils (furuncles), cellulitis folliculitis, carbuncles, scalded skin syndrome, and abscesses, to life-threatening diseases such as pneumonia, meningitis, osteomyelitis, endocarditis, toxic shock syndrome (TSS), bacteraemia and sepsis, respiratory disease (*e.g.*, sinusitis) and food poisoning. It is still one of the five most common causes of nosocomial infections, and is often the cause of postsurgical wound infections.

### Preparation of nutrient agar

Nutrient agar is frequently used for the isolation and purification of bacterial cultures. It can also be used as a means for producing the bacterial lawns needed for antibiotic sensitivity tests. In actuality, antibiotic sensitivity testing is typically performed on media specially formulated for that purpose. Nutrient agar powders of 28 g were suspended in 1 L of distilled water. This mixture was heated while stirring to fully dissolve all components. The dissolved mixture was autoclaved at 121 °C for 15 minutes. Once the nutrient agar was autoclaved, it was then allowed to cool. Then, the nutrient agar was poured into each plate and the plates were left on sterile surface until the agar was solidified. The lid of each Petri dish was replaced, and the plates were stored in a refrigerator.

### Cell growth

*E. coli* (strain of JM103) and *S. aureus* were cultured in Luria Broth (LB, HiMedia) and incubated overnight at 37 °C (at 225 RPM in a shaker incubator). The culture was then centrifuged and the supernatant was aspirated. The cell pellets were uniformly smeared in Nutrient Agar plates (HiMedia) in a laminar hood to avoid contamination. The plated *E. coli* and *S. aureus* were cultured overnight in an incubator at 37 °C to form single colonies. *E. coli* colonies and *S. aureus* colonies were scraped off the LB agar plate using the aseptic technique.

### Antibacterial study of (1 − *x*)BiFeO_3_–*x*NaNbO_3_ powder samples by well diffusion method

**Fig. 8:**
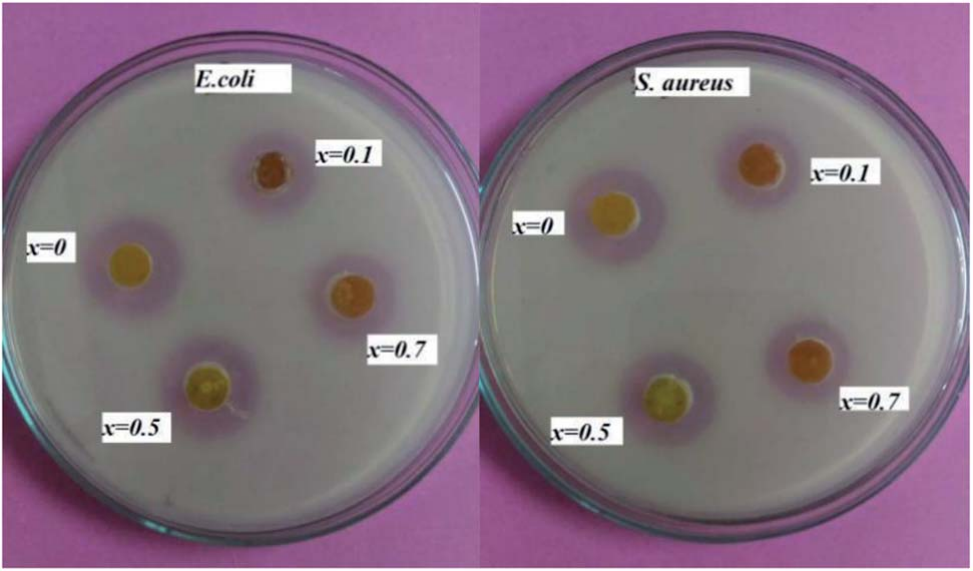
Antibacterial activity of (1 − *x*)BiFeO_3_–*x*NaNbO_3_ powder samples against (a) *E. coli*, (b) *S. aureus*.

The antimicrobial activity of the powder samples assessed using the well diffusion method is shown in Fig. 8. Nutrient agar was poured into the assay plate and allowed to cool down. Once the medium had solidified, three wells (each 1 cm in diameter) were cut out of the agar, and 10 μL of the sample solution were placed into each well. A total of four compounds (*x* = 0, *x* = 0.1, *x* = 0.5 and *x* = 0.7) were placed into each plate, and incubated at 37 ± 0.5 °C for 18 hours. The zone of inhibition was observed. Results showed that all of the compositions were good at bacterial killing. The zone size slightly differed for each compound. The diameter of the zone varied from 0.9 cm to 1.6 cm.

Our composite nanoparticles shows the antibacterial activity against both Gram-negative *E. coli* and Gram-positive *S. aureus*. Compared to the previous reports of the antibacterial activity of Li-doped Bi_0.5_Na_0.45_K_0.5_TiO_3_–BaTiO_3_ ferroelectric material,^7^ our composite showed better results. The material is less toxic in lower concentration. The metal oxide nanoparticle has gained much attention in the medical field as well. BiFeO_3_ is a material that has been widely used for medical application.^24^ Various scientific studies account for the beneficial use of metal nanoparticles to reduce the number of contact-mediated infections.^48^

The direct contact between the bacteria and nanoparticle is an important factor in contact killing.^48^ The free ions from the metal atoms interact with the bacterial cell wall, and lead to the destruction of the cell membrane. O^2−^, OH, and Fe^2+^ are the possible free radicals in the case of the present composite material. It is reported that the hydroxyl group and anion radicals destroy the bacterial cell wall from outside, while H_2_O_2_ directly enters the cell and breaks the membrane.^49^

## Conclusion

BiFeO_3_–NaNbO_3_–PMMA composites film samples were found to exhibit better optical limiting property. The photocatalytic activity was evaluated by the degradation of methyl orange (MO) under UV light. The results indicate that the catalyst could exalt the rate of degradation, as the degradation efficiencies of the dyes are higher with the presence of the catalyst in the given time. The rate of degradation activity is higher for *x* = 0.1 composition. The particle size was found to have an impact on the photocatalytic activity of the material. The enhanced photocatalytic activity of the samples could be attributed to the nano size of the material. The antibacterial activity of the ceramic powder and ceramic-polymer film samples against Gram-negative *E. coli* and Gram-positive *S. aureus* were tested, and it was found that the material is antibacterial and could be potential candidates for biomedical applications. The composition *x* = 0.1 was found to have high antibacterial activity compared to other compositions. This material can be used for tiles, other construction materials and coatings for hospitals and laboratories. It can also be used for water purification to remove toxic organic and inorganic materials in air or solution.

## Conflicts of interest

There are no conflicts to declare.

## Supplementary Material
